# Prognostic Value of PSMB5 and Correlations with LC3II and Reactive Oxygen Species Levels in the Bone Marrow Mononuclear Cells of Bortezomib-Resistant Multiple Myeloma Patients

**DOI:** 10.3390/cimb47010032

**Published:** 2025-01-06

**Authors:** Eva Plakoula, Georgios Kalampounias, Spyridon Alexis, Evgenia Verigou, Alexandra Kourakli, Kalliopi Zafeiropoulou, Argiris Symeonidis

**Affiliations:** 1 Hematology Division, Department of Medicine, School of Health Sciences, University of Patras, 26504 Patras, Greece; eplakoula@gmail.com (E.P.); spyrosal1@hotmail.com (S.A.); jverigou@gmail.com (E.V.); kzafeirop@upatras.gr (K.Z.); 2 Division of Genetics, Cell Biology and Development, Department of Biology, School of Natural Sciences, University of Patras, 26504 Patras, Greece; gkalampounias@ac.upatras.gr; 3 Department of Hematology, OLYMPION General Hospital, Volou & Meilichou Str., 26443 Patras, Greece; akourakli@gmail.com

**Keywords:** multiple myeloma, proteasome, bortezomib resistance, autophagy, biomarkers, oxidative stress, reactive oxygen species, PSMB5, LC3, BMMCs

## Abstract

Proteasome inhibitors (PIs) constitute the most common type of induction treatment for multiple myeloma. Interactions between the proteasome, autophagy, and reactive oxygen species (ROS) have been shown in the past, thus emphasizing the need for a better understanding of the underlying pathophysiology. For this study, bone marrow mononuclear cells from 110 myeloma patients were collected at different disease stages. PSMB5 and LC3I/II protein levels were determined using Western blot, proteasome proteolytic activity (PPA) with spectrofluorometry, and ROS with flow cytometry. PSMB5 accumulation was found to diminish after PI treatment (*p*-value = 0.014), and the same pattern was observed in PPA (*p*-value < 0.001). Conversely, LC3II protein levels were elevated at both remission and relapse compared to baseline levels (*p*-value = 0.041). Patients with a baseline PSMB5 accumulation lower than 1.06 units had longer disease-free survival compared to those with values above 1.06 units (12.0 ± 6.7 vs. 36 ± 12.1 months; *p*-value < 0.001). Median ROS levels in plasma cells were significantly higher at relapse compared to both baseline and remission levels (*p*-value < 0.001), implying poor prognosis. Overall, post-treatment PSMB5 reduction could indicate a shift from proteasomal to autophagic degradation as a main proteostatic mechanism, thus explaining resistance. The elevated oxidative stress in PI-treated patients could possibly serve as an additional compensatory mechanism.

## 1. Introduction

Multiple myeloma (MM) is a mature plasma cell malignancy characterized by the accumulation of abnormal plasma cells in the bone marrow, the induction of osteolytic bone disease, and, in most cases, the production of an abnormal monoclonal antibody (immunoglobulin), which is also commonly referred to as a paraprotein. MM is the second most common hematological cancer (following Acute Myeloid Leukemia, AML) and accounts for more than 10% of all hematological malignancies and for 1% of all types of human cancer [1,2,3]. Its estimated annual incidence rate is about 5 new cases per 100,000 inhabitants, showing a gradual but clearly increasing trend over the last decades, which is estimated to increase even more in the next twenty years [1,4,5]. Even though patients around 65 years of age are mostly affected, it has a rather broad age distribution, being more common among males and people of African descent [6,7,8]. Furthermore, there appears to be a genetic connection to the disease, but our understanding of its hereditary mechanisms remains incomplete [9]. As indicated by its name, MM is characterized by multiple highly vascularized intraosseous tumors that, in the later stages of the disease, may spread through the patient’s body. These metastatic events ultimately manifest various localizations of extramedullary disease [10,11]. Despite the substantial improvement in the understanding of different aspects of MM pathophysiology and the continuous development of novel pharmaceutical agents, mainly targeted types of treatment, MM currently has a 5-year median overall survival (OS) rate of about 55–60% [4]. Besides the conventional treatment options, namely chemotherapy and radiotherapy, immunotherapies, cell therapies, and targeted therapies have also been employed in an effort to improve patients’ disease-free survival (DFS), OS, and quality of life (QoL). Proteasome inhibitors (PIs) constitute the most commonly used type of targeted therapy, approved for the treatment of MM, that has shown increased efficiency and has contributed to the prolongation of life expectancy in newly diagnosed myeloma patients [12,13]. While they have also been used in numerous clinical trials as anti-neoplastic agents for other hematological malignancies and solid tumors [14,15,16,17,18,19], a major burden remains the emergence of resistance against them [12,20,21].

PIs target the ubiquitin–proteasome system (UPS), which is a crucial component of cellular homeostasis in eukaryotic cells being responsible for the degradation of excessive, abnormal, or dysfunctional intracellular polypeptides [22]. Besides the removal of dysfunctional proteins, proteasomal degradation has also been documented to act as an important regulator of many proteins’ turnover rates, thus controlling functions such as signaling cascades, cell cycle progression, and cell death [23,24,25]. More than 80% of the intracellular proteins enter the UPS machinery, thus emphasizing the importance of this system’s integrity in the cell lifecycle [26]. The 26S proteasome is a hollow complex comprising (a) two regulatory subcomponents (19S proteasomes) bound on the ends of the barrel-like core that recognize the polyubiquitinated polypeptides and (b) the catalytic core, which acts as an ATP-dependent proteolytic subcomponent (20S proteasome) [22]. More precisely, β1 (20S proteasome subunit beta-1 or proteasome subunit beta type-6, PSMB6), β2 (20S proteasome subunit beta-2 or proteasome subunit beta type-7, PSMB7), and β5 (20S proteasome subunit beta-5, or proteasome subunit beta type-5, PSMB5) subunits of the 20S proteasome contain active sites with proteolytic specificities, which constitute caspase-like (C-L), trypsin-like (T-L), and chymotrypsin-like (ChT-L) activity, respectively [22,27].

Malignant cells rely heavily on the uncompromised function and integrity of the UPS due to their increased metabolic, biosynthetic, and proliferation rates, all of which lead to an accumulation of abnormal, misfolded, or simply excessive proteins and an increased need for maintenance and fine-tuning of intracellular proteostasis [13,28,29,30,31]. In many types of cancer cells (including MM), elevated 26S proteasome, as well as increased proteasome proteolytic activity (PPA), have been reported [32,33,34]. In breast cancer cells, mean PPA was measured at 144 ± 101 units, while in 25 other cancer specimens, it was found to be 699 ± 489 units [14]. Kumatory et al. showed that in four neoplastic hematopoietic cell lines, the proteasome content was about four times higher than the corresponding normal mononuclear cells [33]. In colon cell lines, a strong S5a and a-5 subunit expression has been detected, which was associated with higher PPA [34]. It has also been shown that MM cells and pancreatic cancer cells, which are characterized mainly by secreting activity, are greatly dependent on the ubiquitin–proteasome pathway [35,36]. Bortezomib (Velcade^®^ or PS-341), the first PI approved as an anticancer agent, inhibits the ChT-L proteasome activity and, more specifically, the action of the PSMB5 and the PSMB1 20S proteasome subunits [37]. As a result, the accumulation of an excessive intracellular proteinic load, which would otherwise trigger the unfolded protein response (UPR) and induce endoplasmic reticulum (ER) stress and apoptotic cell death [38]. Carfilzomib (Kyprolis^®^) was later discovered and was introduced in clinical practice as a more potent PI since it binds irreversibly to the active proteasomal sites and leads not only to transient proteasome inhibition and impaired proteostasis but, on top of that, to permanent stultification and proteasomal degradation [39]. Additional PIs have later been discovered and have been used at the preclinical or investigational level, whereas a third agent, Ixazomib (Ninlaro^®^), which is orally available, has achieved approval and is also used in clinical practice [40]. Resistance following exposure to PIs is a major setback for treatment; it is not entirely understood, and many mechanisms have been proposed to explain it [12,41,42,43]. Mutations in the *PSMB5* gene were the first plausible explanations since the selection pressure and bottleneck events lead to the survival of clones carrying PI-resisting PSMB5 mutations [44,45,46,47,48]; nonetheless, PI resistance, not associated with genetic alterations, has also been reported [11,49]. Ever since the first observations of resistance, multiple potentially implicated mechanisms have been proposed. In particular, alterations in signaling pathways controlling stress response, survival, and evasion of apoptosis have been reported [42,50]. The main examples are altered NF-κΒ, MAPK, and Akt/mTOR signaling; cell cycle regulation through p21 and p53; heat shock protein expression (mainly Hsp70); and antiapoptotic signaling through Noxa/Bcl-2 activation [42,43,50]. Additionally, at least to some extent, multidrug resistance (MDR) has also been reported as a mechanism of Bortezomib-induced cell death evasion [50]. Although the main mechanism of MDR contributing to Bortezomib resistance is the drug efflux activity, MDR remains a minor contributor to PI resistance [50]. Most reported mechanisms are focused on the bypassing of PI-imposed proteasome blockage and apoptosis activation.

Autophagy, another major proteolytic pathway, may act as a pro-survival mechanism by counterbalancing proteasome insufficiency, potentially resulting in the protection of the cells from apoptotic death [51,52,53]. Autophagy and UPS-dependent degradation act as synergistic mechanisms, physiologically possessing slightly different roles [52,54]. While the UPS is responsible for the degradation of most intracellular proteins (once they have been labeled for degradation), autophagy acts on aggregated proteins and on bigger structures (even organelles) [54]. The two pathways often complement each other when intracellular stress levels are high, and an excessive need for protein recycling or nutrients is necessary. The main linking molecules are molecular chaperones that direct ubiquitin-labeled polypeptides to the autophagosomes and transcription factors that co-activate the two distinct pathways. Several groups have reported that autophagy is associated with PI resistance in both hematological malignancies and solid tumors [43,55,56]. Our research team has recently highlighted the mechanisms of PI resistance in solid tumors, particularly in prostate cancer cell lines, in which autophagic markers, such as LC3 (microtubule-associated proteins 1A/1B light chain 3A, MAP1LC3A, or ATG8E), were found to be significantly upregulated in Bortezomib-resistant cells [43,57]. Autophagy and apoptosis are regulated by a dynamic balance inside tumor cells, an axis that regulates the intracellular stress levels and increases or decreases the activity of self-digesting pathways in order to restore impaired cell homeostasis [51]. A potential role of autophagy in PI resistance of MM cells could reinforce our understanding of the phenomenon and provide novel therapeutic targets. Besides autophagy in PI resistance, a significant role is attributed to the role of oxidative stress [41,43,57,58]. More specifically, in many types of cancer, chemotherapy is known to further increase endogenous reactive oxygen species (ROS) levels, as this has been reported for MM following treatment with Bortezomib [31,58,59,60]. Particularly in MM cells, in which immunoglobulin rapid synthesis and storage is taking place, ROS synthesis is largely elevated [60]. Cancer cells inherently generate intracellular ROS to a higher level compared to normal cells, which is attributed to their higher metabolic rate, hypoxic conditions due to the defective vasculature, and/or potentially attributed to specific gene mutations [61,62]. Increased ROS accumulation in a cell leads to increased intracellular oxidative stress and subsequently to the induction of ferroptotic or apoptotic cell death. Additionally, excessive ROS is a trigger for autophagy activation, which is regarded as a rescue mechanism that attempts to reduce the circulation of oxidatively damaged molecules that lead to apoptosis [63]. Therefore, closely monitoring ROS levels and purposefully administering oxidative-stress-inducing agents could increase the cell susceptibility to cell death mechanisms and thus be used in the treatment of MM and other neoplastic diseases [61,62,64].

This study investigates the potential association between proteasomal activity in the bone marrow mononuclear cells of a cohort of MM patients (stratified according to International Staging System {ISS} disease status) and alterations in their autophagic activity, as well as with the generation of ROS, with the purpose of highlighting novel potential prognostic factors and therapeutic targets for these patients. Proteasome activity and basic autophagy and stress markers can be easily assessed from routinely obtained bone marrow samples and thus provide significant insight regarding changes in the bone marrow microenvironment. This type of information can assist the treating physicians in better estimating the condition of each patient and the effectiveness of treatment. Additionally, further disambiguation of the interplay between those mechanisms is expected to expand our current armory against MM (and probably also related to hematological malignancies) and pave the road for a better understanding of drug resistance development and development of possible prevention/reversal strategies.

## 2. Materials and Methods

### 2.1. Patient Inclusion Criteria

For this study, 110 MM patients, diagnosed, treated, and followed up at the Department of Hematology of the University Hospital of Patras, were included. The applied treatment combinations consisted of the following regimens which contained PIs PIs: Bortezomib–Cyclophosphamide–Dexamethasone (VCD), Bortezomib–Lenalidomide–Dexamethasone (VRD), Bortezomib–Melphalan–Prednisone (VMP), Daratumumab–Bortezomib–Melphalan–Prednisone (DVMP), Bortezomib–Dexamethasone (Vel/Dex), Bortezomib–Adriamycin–Dexamethasone (PAD), and Cyclophosphamide–Adriamycin–Dexamethasone (CAD). Patients at different disease stages were selected to represent those at baseline (initial diagnosis), those at their first remission, and those at their first relapse. Since bone marrow samples from healthy individuals were not available, 17 patients with (newly diagnosed) early-stage (I–IIA) Diffuse Large B-Cell Lymphoma (DLBCL) were included and were used as controls. To confirm that bone marrow of the 17 DLBCL patients was not involved by lymphomatous cells, a baseline PET/CT (Positron Emission Tomography–Computed Tomography and a bone marrow trephine biopsy were performed. Both tests demonstrated no disease infiltration at the point of sample collection, thus allowing their use as controls. All subjects tested provided written informed consent before participating in the study. The study protocol was approved by the Ethical and Scientific Committee of the University Hospital of Patras (Approval Number: 241/16.06.20; see relevant information in the Informed Consent Statement section).

### 2.2. Sample Collection

A sample of 10 mL of whole bone marrow aspirate was used for all analyses. Bone marrow mononuclear cells (BMMCs) were obtained following density gradient centrifugation of the aspirates at 1600 rpm for 30 min by using 3 mL of Lymphosep™ (Cat. No. L0560, Biowest, Nuaillé, France). The cells were then rinsed with a sterilized PBS solution and aliquoted to be used in downstream analyses. Τhe MM patients used for the study were screened for bone marrow infiltration by flow cytometry (using the CD138+/CD38+ markers).

### 2.3. Proteasome Proteolytic Activity Assay

To study PPA, focus was given on the main catalytic subunit of the 26S proteasome, the PSMB5 (or β5 subunit), which possesses chymotrypsin-like activity (ChT-L) that can be quantified with spectrofluorometry (fluorescence spectroscopy). BMMCs were analyzed using a solution containing 1 M of 1,4-Dithiothreitol (DTT) (Cat. Number 10197777001, Merck, Darmstadt, Germany), as described in other publications [44,65]. Total protein was extracted from the cells, and the protein concentration was estimated using Q5000 Nanodrop Quawell (LabWrench, San Diego, CF, USA). Equal amounts of extracted protein from each sample were incubated with the proteasome fluorogenic substrate-peptide LLVY-AMC (Suc-Leu-Leu-Val-Tyr-7-amide-4-methylcoumarin) (Cat. Number 3120-v, Peptide Institute Inc., Osaka, Japan) for 1 h in the dark, at a temperature of 37 °C. The proteasome inhibitor MG-132 (Cat. Number 3175-v, Peptide Institute Inc., Osaka, Japan) was used as a control for the assay. Subsequently, PPA was estimated via fluorescence emission at 440 nm, following excitation of the fluorophore at 373 nm, using a Tecan Magellan™ spectrometer (Tecan, Männedorf, canton of Zürich, Switzerland). Mean values from triplicate measurements were collected, the obtained fluorescent intensity values were divided by each sample’s mass (as had been determined using spectrophotometry), and the results were presented as “mean intensity per mg of total protein”. Data analysis was performed using the SPSS^®^ software (version 29.0.0) (IBM^®^, Armonk, NY, USA).

### 2.4. Western Blot Analysis

To study the accumulation of proteasome- and autophagy-related markers, Western blot analysis was performed. Protein was extracted from the BMMCs using a Western blot (WB) lysis buffer with a pH = 8 containing 10 mM Tris-HCl, 1 mM EDTA, 1% Triton X-100, 0.1% sodium deoxycholate (NaDoC), 140 mM NaCl, 0.1% sodium dodecyl sulfate (SDS), 1 mM NaF, and 0.5 mM Na_3_VO_4_, all obtained from Merck (Merck, Darmstadt, Germany). The protease inhibitors cocktail was obtained by Thermo Fisher Scientific (Waltham, MA, USA) and was used in the concentration suggested by the manufacturer. The final protein concentration of the lysates was estimated using Q5000 Nanodrop Quawell (LabWrench, San Diego, CF, USA), and 50 μg of each sample was loaded on 12% SDS-polyacrylamide gels. All electrophoresis consumables were purchased from Merck (Darmstadt, Germany). Following SDS-PAGE (sodium dodecyl sulfate–polyacrylamide gel electrophoresis), semi-dry electrotransfer of the proteins was performed using the Towbin’s buffer for 30 min on an Immobilon-P^®^ PVDF 0.48 μm membrane from Sigma-Aldrich (Darmstadt, Germany). As a blocking solution, 5% skimmed milk was used in a Tris-Buffered Saline (TBS buffer) containing 0.1% Tween^®^ 20 (Merck, Darmstadt, Germany), and the membranes were blocked for 1 h at a temperature of 37 °C. The antibodies used were a mouse polyclonal Anti-PSMB5 IgG antibody (Ab)(sc-393931, Santa Cruz Biotechnology, Dallas, TX, USA) diluted by 1:1000 for proteasome detection, a rabbit polyclonal Anti-LC3I/II IgG Ab (Cat. Number 4108, Cell Signaling Technology, Danvers, MA, USA) diluted by 1:1000 for the autophagy process detection, and a rabbit monoclonal anti-beta-actin IgG Ab (Cat. Number sc-47778, Santa Cruz Biotechnology, Dallas, TX, USA) diluted by 1:2000 as a loading control. As secondary antibodies, an Anti-mouse IgG HRP-linked (horseradish peroxidase-linked) Ab (Cat. Number 7076S, Cell Signaling Technology, Danvers, MA, USA) and an Anti-rabbit IgG HRP-linked antibody (Cat. Number 7074S, Cell Signaling Technology, Danvers, MA, USA) were used, both of which were diluted by 1:2000. All antibodies were diluted in a TBS buffer containing 5% skimmed milk and 0.1% Tween^®^ 20 (Merck, Darmstadt, Germany), all primary Ab incubations were performed at 4 °C overnight, and all secondary HRP-linked Ab incubations were performed at 37 °C for 1 h. The SuperSignal™ West Pico PLUS Chemiluminescent Substrate was used for antigen detection (Thermo Fisher Scientific, Waltham, MA, USA), and autoradiography films (Fujifilm Hellas, Athens, Greece) were used to develop the signal.

### 2.5. Western Blot Data Quantification

To obtain quantitative data from WB, protein samples were loaded on the same SDS-PAGE. Optimization of the loaded protein’s amount was performed prior to each analysis to avoid signal saturation. To verify the process, beta-actin was also detected using a specific Ab. Overexposed films were avoided and omitted from the analytical procedure, and films in which no visible oversaturation was present were scanned. All scanned data were transformed into grayscale images using FIJI (version 2.9.0) [66]. To obtain information about the signal intensity of each blot, the ImageJ/FIJI plug-in Gel Blot was used [67]. Each sample was analyzed in triplicate using WB, and the mean intensity values were used for the subsequent statistical analyses. To compare the different patients, normalization was performed, using beta-actin intensity to diminish possible slight differences in the arbitrary units, and produced by the digital tool. Mean values from triplicate measurements were collected, the obtained chemiluminescence intensity values were divided by each sample’s mass (as had been determined using spectrophotometry), and the results were presented as “mean intensity per mg of total protein”. Data analysis was performed using SPSS^®^ software (version 29.0.0) (IBM^®^, Armonk, NY, USA).

### 2.6. Flow Cytometry

Flow cytometry was used to separate the BMMCs into subpopulations and measure their intracellular ROS level. The subsequent antibodies conjugated to fluorophores were used: mouse Anti-CD138 IgG linked to allophycocyanin (Anti-CD138-APC) (clone MI15- 347216, BD Biosciences, Franklin Lakes, NJ, USA) as a myeloma marker, a mouse Anti-CD38 IgG phycoerythrin (PE) linked (Anti-CD38-PE) (clone HB-7, BD Biosciences, Franklin Lakes, NJ, USA) to evaluate disease aggressiveness, and a mouse Anti-CD34 linked to phycoerythrin-cyanine5 (Anti-CD34PeCy5) (clone 581, BD Biosciences, Franklin Lakes, NJ, USA) as a lymphohematopoietic stem cell marker. To measure the intracellular accumulation of ROS in BMMCs, a chemically reduced form of fluorescein and hydroxyl radical marker 2′,7′-dichlorodihydrofluorescein diacetate (H_2_DCFDA) (Cat. Number 18825580, Invitrogen, Waltham, MA, USA) was used [43]. Flow cytometry analysis was performed using the BD FACS Calibur™ cell cytometer (BD Biosciences, Franklin Lakes, NJ, USA), and 400,000 cells were acquired per sample for the analysis. The FlowJo™ Software (v.10.0.6) (BD Biosciences, Franklin Lakes, NJ, USA) was used to interpret and plot flow cytometry data.

### 2.7. Statistical Analysis and Data Plotting

Statistical analysis was performed using the SPSS^®^ IBM^®^ statistics package (version 29.0.0) (IBM^®^, Armonk, NY, USA). All continuous variables (e.g., ROS accumulation, PSMB5 accumulation levels) were analyzed using the descriptive statistics functions. To compare the means of two groups, independent sample *t*-tests were used for patients belonging to different disease stages (baseline, remission, or relapse strata). Paired *t*-test was used to analyze matched pairs of samples (baseline vs. remission or baseline vs. relapse). One-way analysis of variance (ANOVA) was used to compare multiple groups. Statistical significance level was set to 5%. To estimate the strength of linear relationships between continuous variables (PSMB5, LC3 accumulation, PPA, ROS levels) in each of the three MM patient groups (baseline, remission, and relapse), Pearson’s correlation was used. Positive r values indicated a positive correlation, while negative r values indicated a negative correlation. Survival analyses were performed in order to estimate the DFS using the Kaplan–Meier curves. All plots within this article were created in Prism 8 (version 8.4.3) (GraphPad, La Jolla, CF, USA) from the data obtained from the SPSS^®^ analyses.

## 3. Results

### 3.1. Patient Demographics and Baseline Clinical Characteristics

Patients’ median age was 64 years (Interquartile range, IQR = 15); 45 of them (40.5%) were female, and the remaining 65 were male (59.5%). Their main clinical characteristics are presented in Table 1. Sixty-one (61) patients (55.4%) had IgG-, 29 (26.4%) had IgA-, and the remaining 20 (17.2%) had light-chain myeloma. Disease classification according to ISS revealed stage I for 34 patients (30.9%), stage II for 28 (25.5%), and stage III for the remaining 46 (43.6%). Moreover, 22 out of 97 patients tested (20% of the total patient sample) exhibited poor-prognosis cytogenetic abnormalities (estimated by fluorescent in situ hybridization {FISH}), 10 of them (9%) had renal impairment, 42 (38%) had anemia, and 74 (67.2%) had osteolytic bone disease (Table A1).

Patients were stratified into three groups, depending on their disease status at the time of evaluation. Thus, 31 patients were studied at initial diagnosis (baseline, pre-treatment), 36 patients were studied at their first remission (≥VGPR {Very Good Partial Response} according to IWG {International Working Group} criteria), and the remaining 43 were studied at their first relapse. Each stratum contains unrelated patient samples. For a smaller number of patients, data were available at two time points: initial diagnosis and at their first remission (*n* = 7) or their first relapse (*n* = 7). These samples (being paired samples) were analyzed independently from the remaining, and the results obtained are presented separately in Section 3.4. The seven patients studied at baseline/remission were named the Remission Cohort, and the seven patients studied at baseline/relapse were named the Relapse Cohort.

For this study, total BMMCs were used, which were obtained after separation from whole bone marrow aspirates. A fraction of each sample was stained with Anti-CD138 and Anti-CD38 Abs, and using flow cytometry, the plasma cell (CD138+/CD38+) percentage of each sample was determined. As shown in Table 1, patients at baseline were found to have 35–60% plasma cells. Patients during first remission exhibited a significant reduction, and only 1–5% of total BMMCs were plasma cells; patients at their first relapse had 15–35% CD138+/CD38+ plasma cells among their total BMMCs. The control group was confirmed to have no bone marrow plasma cell infiltration, with only 0–1% of total BMMCs being double positive from plasma cell markers. Therefore, the use of this group was justified as a control, and their non-infiltrated marrow status was confirmed.

### 3.2. Proteasome Activity Diminishes Following Treatment with Proteasome Inhibitors Both at Remission and at Relapse

**Figure 1:**
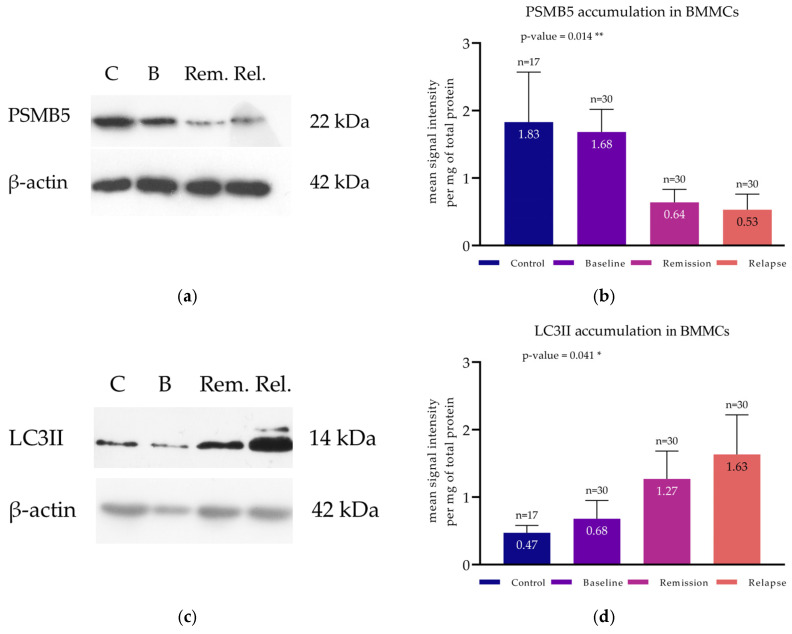
Protein accumulation assay of PSMB5 and LC3II in BMMCs. (**a**,**c**) Western blot analysis for the detection of PSMB5 and LC3II in the BMMCs of representative MM patients at baseline, first remission, and relapse and of controls. Beta-actin was used as a loading control to verify equal protein mountings. All immunoblotting experiments were conducted in triplicate for each subject (*n* = 110). (**b**,**d**) Protein levels of PSMB5 and LC3II of all available MM patients at baseline (*n* = 30), first remission (*n* = 30), and relapse (*n* = 30) and of controls (*n* = 17). Each bar corresponds to the mean signal intensity per mg of total protein; the error bars correspond to the standard deviation. * to a *p*-value < 0.05; ** corresponds to a *p*-value < 0.01.

Proteasome proteolytic activity was studied at the protein level by assessing the intracellular concentration of the main catalytic subunit bearing ChT-L (PSMB5) with WB analyses (Figure 1a) and the enzymatic activity levels by evaluating the proteasome’s efficiency to hydrolyze specific substrate with fluorescence spectrometry.

#### 3.2.1. PSMB5 Determination

Regarding PSMB5, its mean intracellular concentration was not significantly different when the whole group of 110 patients and the controls were compared (1.58 ± 1.17 versus 1.83 ± 0.74, respectively, *p*-value = 0.131). Conversely, by comparing patients’ PSMB5 levels according to disease status, significant differences were observed. Patients studied at baseline had significantly higher PMB5 levels (1.68 ± 0.34) compared to those studied at remission (0.64 ± 0.19, *p*-value = 0.042) (Figure 1b) and at relapse (0.53 ± 0.23, *p*-value = 0.039). On the other hand, no significant difference between MM patients studied at baseline and controls was found (1.68 ± 0.34 versus 1.83 ± 0.74, *p*-value = 0.260). Of interest, patients studied either at remission or at relapse exhibited significantly lower PSMB5 protein levels compared to controls (*p*-value = 0.031 and *p*-value = 0.024, respectively). One-way ANOVA revealed that PSMB5 levels across all patient groups were significantly different (F [3, 123] = 104.8, *p*-value = 0.014). These results are shown in Figure 1a,b.

#### 3.2.2. PPA Determination

**Figure 2:**
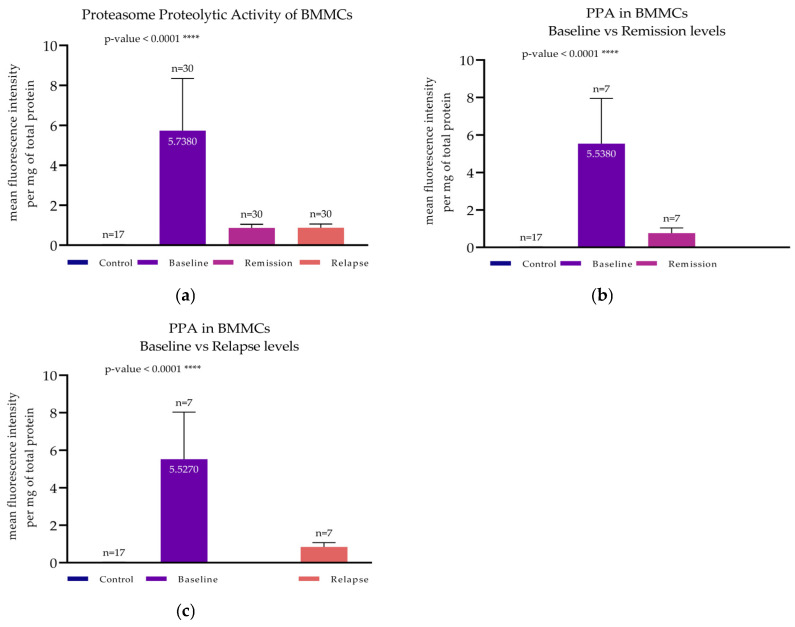
Proteasome proteolytic activity (PPA) determination of BMMCs by spectrofluorometry in MM patients at baseline (*n* = 30), first remission (*n* = 30), and relapse (*n* = 30) and of controls (*n* = 17). (**a**) One-way ANOVA confirmed that all groups exhibited a statistically significant difference (*p*-value < 0.0001). (**b**) PPA of a subgroup of MM patients (*n* = 7) at baseline and at their first remission. Patients in remission exhibited significantly decreased PPA (*p*-value < 0.0001). (**c**) PPA of a subgroup of MM patients (*n* = 7) at baseline and at relapse. Again, patients at relapse showed significantly decreased values (*p*-value < 0.0001). (**a**–**c**) Each bar corresponds to the mean fluorescence intensity per mg of total protein, and the error bars correspond to the standard deviation. **** corresponds to a *p*-value < 0.0001.

Subsequently, the PPA of all groups was determined, and the mean values were compared. Patients’ PPA levels at baseline were significantly higher compared to those of the remaining two patient groups (at remission and at relapse), as well as to controls (*p*-values < 0.0001 for all comparisons). The PPA of untreated MM patients exhibited a two-hundred-fold increase, as compared to controls (5.74 ± 2.61 versus 3.03 × 10^−2^ ± 3.7 × 10^−4^ mean fluorescence intensity per mg of total protein) (Figure 2a). A statistically significant difference was also observed between the PI-treated (Bortezomib) and PI-untreated patients; PPA levels in both patient groups (at relapse and at remission) were closely comparable (0.88 ± 0.18 and 0.87 ± 0.17 mean fluorescence intensity per mg of total protein, respectively), as is also shown in Figure 2a.

#### 3.2.3. Linear Correlation Between PSMB5 Accumulation and PPA of Each Stratum

**Figure A1:**
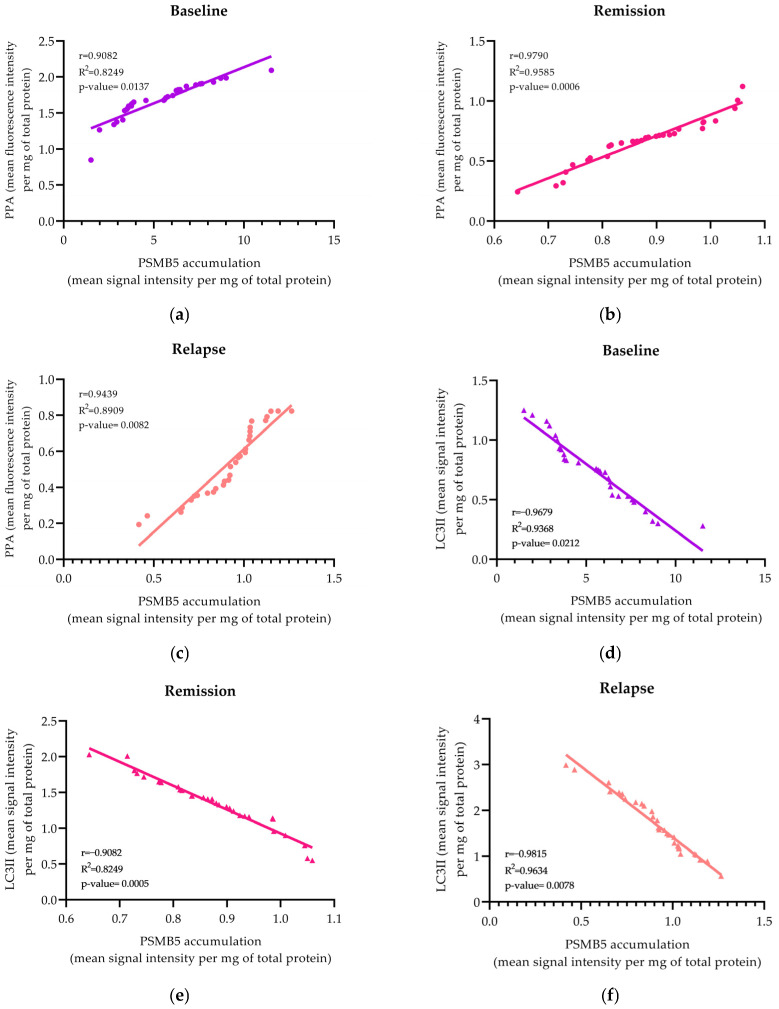
Pearson’s correlations of PSMB5 with PPA/LC3II. (**a**) PSMB5 and PPA coefficient at baseline, (**b**) at remission, and (**c**) at relapse. (**d**) PSMB5 and LC3 at baseline, (**e**) at remission, and (**f**) at relapse. (**a**–**f**) Pearson’s coefficient is annotated as (r), the correlation coefficient is annotated as (R^2^), and the *p*-values correspond to the linear regression analysis.

Finally, the potential association between PSMB5 protein levels and the corresponding PPA of each sample was examined using Pearson’s correlation coefficient for each of the three patient strata (baseline, remission, relapse) (Figure A1a–c). Indeed, a very strong positive correlation was revealed in all patient groups (baseline: r = 0.908, *p*-value = 0.014; remission: r = 0.979, *p*-value < 0.001; relapse: r = 0.944, *p*-value = 0.008), suggesting that the elevated proteasome subunit protein levels directly led to a linear increase in the PPA.

### 3.3. LC3II Protein Levels Keep Increasing During Remission and Peak at Relapse

#### 3.3.1. LC3II Determination

Autophagy mostly affects the disintegration of damaged subcellular organelles and polypeptides, the subsequent recycling of their amino acids, and the regulation of various proteins, whose concentration is tightly regulated by the cell mechanisms’, using targeted proteolysis. In cases of an impaired UPS, main autophagy proteins (molecular chaperones, mediators, and effectors) can be upregulated to substitute for the inactivated proteasomes, thus accelerating the autophagic flux. LC3 is a major autophagic protein, which can be used to monitor this process and provide information about the pathways’ activity (Figure 1c).

Patients tested at baseline and controls presented significantly lower mean LC3II protein expression levels compared to patients belonging to the remaining two groups. These differences were statistically significant with *p* = 0.041 (Figure 1d).

#### 3.3.2. Association Between PSMB5 and LC3II Accumulation

To investigate a potential association between autophagy and UPS at the protein level, the Pearson correlation coefficient between the two sets of values was calculated for each stratum. PSMB5 subunit levels and LC3II expression were negatively correlated to a statistically significant degree in all strata. Patients at baseline exhibited a negative correlation with an r = −0.968 and *p*-value = 0.021 (Figure A1d). Patients at remission had an r = −0.908 and a *p*-value = 0.0005 (Figure A1e), and patients at relapse had an r = −0.982 and a *p*-value = 0.008 (Figure A1f). These findings imply the presence of a potential interplay between the UPS system and autophagy in myelomatous cells, leading to the inhibition of proteasome formation, most probably resulting from the autophagic destruction of its proteolytic subunits, such as the β5 subunit.

### 3.4. Oxidative Stress Levels in the BMMCs of Multiple Myeloma Patients

To study the oxidative condition of the cells, we focused on assessing the intracellular ROS levels, as well as a major enzyme that mitigates ROS-induced damage, human manganese superoxide dismutase (MnSOD).

#### 3.4.1. Determination of Intracellular Reactive Oxygen Species Levels in Total BMMCs

**Figure 3:**
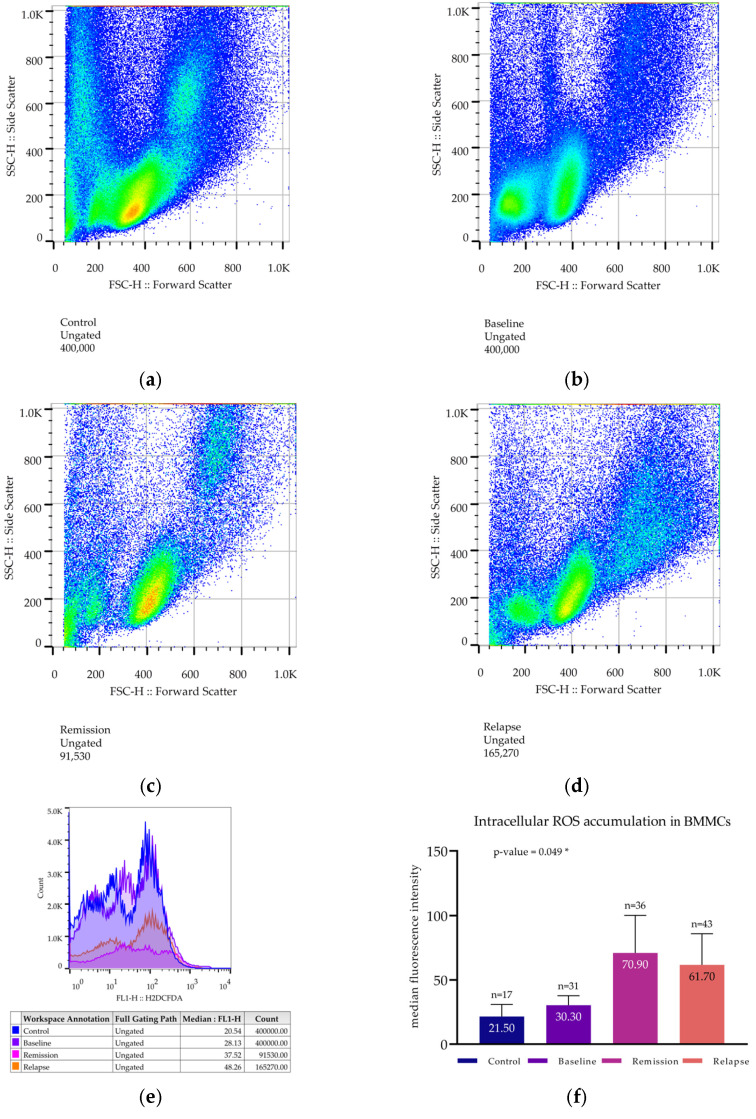
Estimation of reactive oxygen species levels. BMMCs were incubated with H_2_DCFDA. (**a**–**e**) Flow cytometry data of representative patients from each patient group: (**a**) A typical scattergram of BMMCs from a control subject; (**b**) ROS levels of a patient at baseline; (**c**) ROS levels of a patient in remission; (**d**) ROS levels of a patient in relapse. (**e**) Histogram of H_2_DCFDA fluorescence intensity of BMMCs from all groups. (**f**) Bar chart where the mean values from each patient group are compared. Each bar represents the mean of all median values. The number of patients is annotated, as is the sample’s mean. * corresponds to a *p*-value < 0.05.

For ROS levels, Median Fluorescence Intensity (MFI) was used instead of mean intensity in order to exclude outliers and produce better estimations of the population’s true conditions (Figure 3a–e). Intracellular ROS MFI was found higher at both relapsing (61.7 ± 24.3) and the remission group (70.9 ± 29.3) compared to the group studied at baseline (30.3 ± 7.4) and the control group (21.5 ± 9.4). Following one-way ANOVA, the difference was of statistical significance (*p*-value = 0.05) (Figure 3f).

#### 3.4.2. Intracellular Reactive Oxygen Species Determination of CD138+/CD38+ Cells

**Figure 4:**
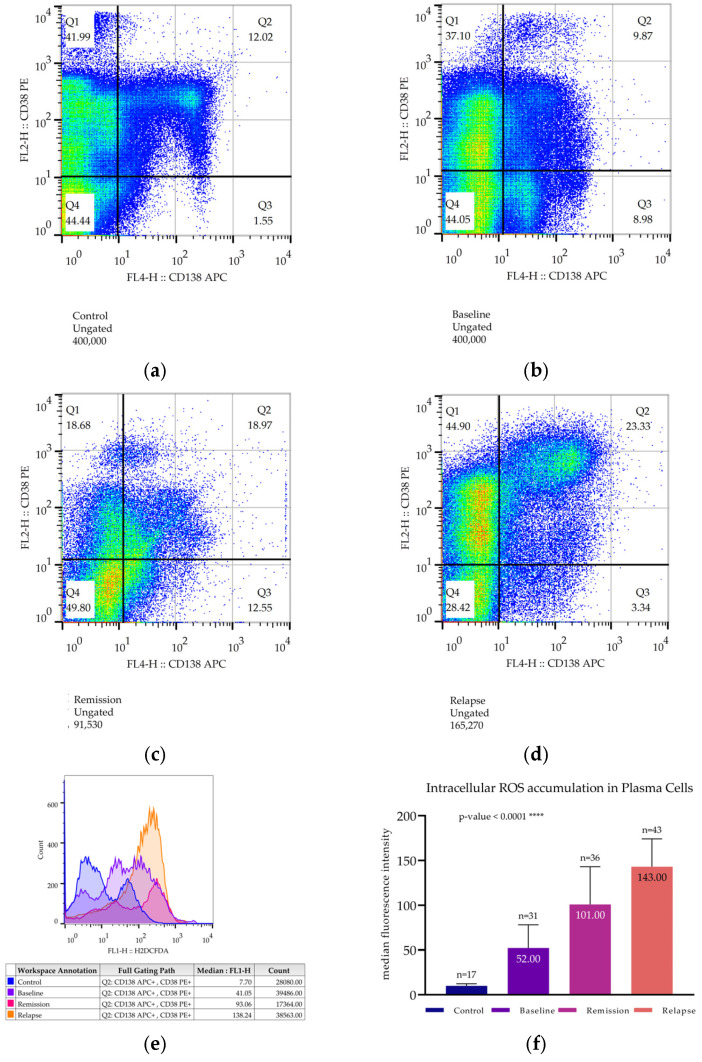
Estimation of reactive oxygen species levels in the CD138+/CD38+ subpopulation (plasma cells) of BMMCs. (**a**–**e**) Flow cytometry data of representative patients from each patient group: (**a**) A typical scattergram of BMMCs from a control subject, (**b**) scattergram of a patient at baseline, (**c**) scattergram of a patient in his first remission, and (**d**) scattergram of a patient during first relapse. (**e**) Histogram of H_2_DCFDA fluorescence intensity of CD138+/CD38+ BMMCs from all groups. (**f**) Bar chart where the mean values from each patient group are compared. Each bar represents the mean of all median values. The number of patients is annotated, as is the sample’s mean. **** corresponds to a *p*-value < 0.0001.

Staining for the surface markers CD138 and CD38 is routinely used to identify plasma cells since only the double-positive cells belong to the myelomatous population (Figure 4a–e). The control group exhibited lower ROS levels (10.1 ± 2.37), followed by MM patients studied at baseline (52 ± 26), whereas MM patients at relapse and those at remission had significantly higher ROS levels (143 ± 31 and 101 ± 42, respectively, *p* < 0.0001 for both comparisons) (Figure 4). As previously described, elevated ROS levels can be attributed to PI exposure, which leads to UPS failure and a subsequent accumulation of free radicals that can propagate oxidative damage throughout the cell. This effect is obvious in BMMCs (Figure 4) but mostly affects CD138+/CD38+ cells since they are more active in terms of protein synthesis due to an overproduction of immunoglobulin chains.

#### 3.4.3. MnSOD Determination

**Figure 5:**
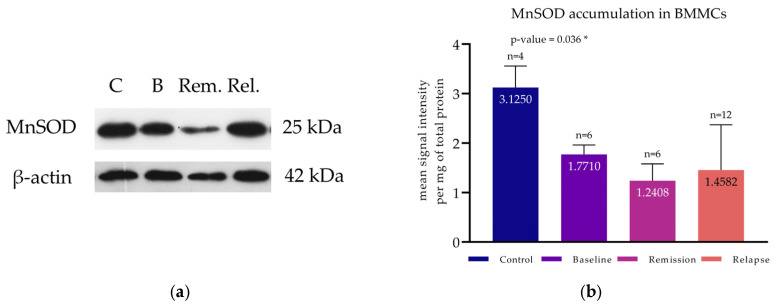
Protein accumulation assay of MnSOD. (**a**) Western blot analysis for the detection of MnSOD in the BMMCs of representative MM patients at baseline, first remission, and relapse and of controls. All immunoblotting experiments were conducted in triplicate for each subject (*n* = 24). (**b**) Protein levels of MnSOD of all available MM patients at baseline (*n* = 6), first remission (*n* = 6), and relapse (*n* = 12) and of controls (*n* = 4). Each bar corresponds to the mean signal intensity per mg of total protein, and the error bars correspond to the standard deviation. * corresponds to a *p*-value < 0.05.

An additional study of the antioxidant capacity of the cells was performed on the total BMMCs of a smaller group of patients (*n* = 24) by assessing the levels of the mitochondrial enzyme MnSOD (or superoxide dismutase 2, SOD2) using WB (Figure 5a).

The control group exhibited higher MnSOD levels (3.13 ± 0.44) compared to baseline levels of MM patients (1.77 ± 0.19, *p*-value = 0.02). Patients at remission had the lowest MnSOD accumulation (1.24 ± 0.34), whereas patients at relapse exhibited slightly higher levels (1.46 ± 0.91) (Figure 5b). In general, multiple myeloma patients had significantly lower MnSOD levels compared to controls (*p*-value < 0.001), as has also been shown by other researchers in the past [68,69].

### 3.5. PSMB5 and LC3II Are Negatively Correlated During Remission and Relapse

For 14 patients, BMMC specimens were available for analysis at two different time points of their disease course. Each patient sample was tested in triplicate. WB was used to determine PSMB5 and LC3II accumulation in order to further standardize the results (expressed in arbitrary units), and beta-actin was used as the normalization protein. In PPA determination, the results were normalized based on the total cellular protein (expressed in mg).

#### 3.5.1. PSMB5 and LC3II at Remission

**Figure 6:**
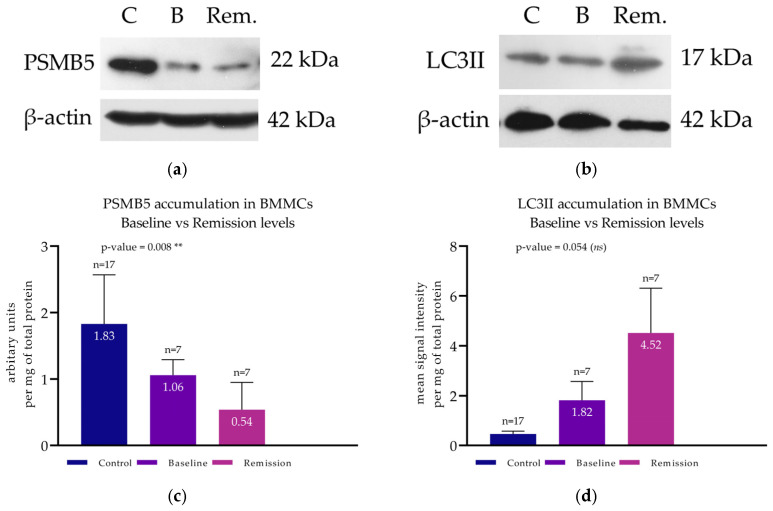
Protein accumulation of PSMB5 and LC3II in BMMCs of patients in remission. (**a**) Western blot analysis for the detection of PSMB5 of a representative MM patient at baseline and at first remission. (**b**) Western blot analysis for the detection of LC3II of a representative MM patient at baseline and at remission. (**c**) Protein levels of PSMB5 at both time points of the whole patient cohort (*n* = 7) and of the controls. (**d**) Protein levels of LC3II at both time points of the whole patient cohort and of the controls. (**c**,**d**) Each bar corresponds to the mean signal intensity per mg of total protein of each patient group, and the error bars correspond to the standard deviation. “*ns*” corresponds to non-significant; ** corresponds to a *p*-value < 0.01.

A first cohort of patients (*n* = 7) (named the Remission Cohort) was formed with paired bone marrow samples, both from the initial diagnosis and the remission stage. In these patients, PSMB5 accumulation was found at 1.06 ± 0.23 at initial diagnosis and 0.54 ± 0.41 at remission (Figure 6a). This difference was statistically significant, using a paired *t*-test, with a *p*-value of 0.008 (Figure 6c). PPA exhibited the same pattern as PSMB5 accumulation in patients in remission (Figure 2b). One-way ANOVA verified the statistical difference (*p*-value < 0.0001). Simultaneous with the PSMB5 reduction, a significant increase in LC3II levels was documented (Figure 6b). Mean LC3II levels at baseline were 1.82 ± 0.75, and at remission, they were 4.52 ± 1.79 (Figure 6d). Paired *t*-test verified the significant difference between the two groups (*p*-value = 0.049) (Figure 6d). Western blots of both PSMB5 and LC3II from representative patients belonging to the Remission Cohort are presented in Figure 6a,b.

#### 3.5.2. PSMB5 and LC3II at Relapse

**Figure 7:**
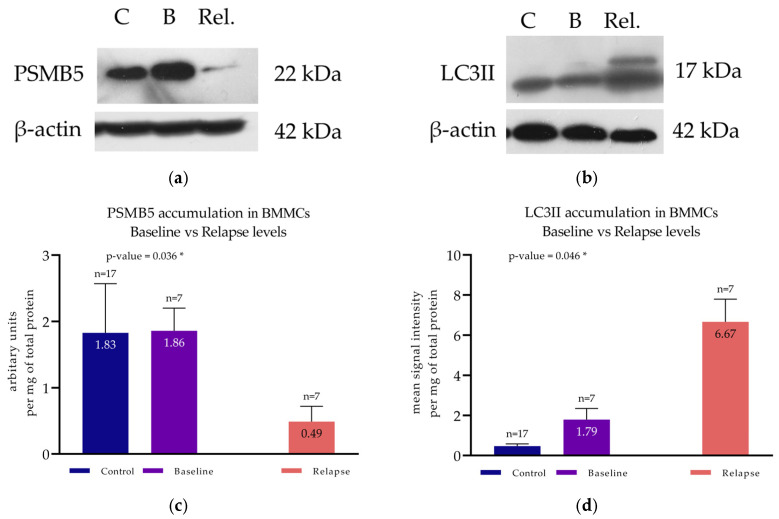
Protein accumulation of PSMB5 and LC3II in BMMCs of patients at relapse. (**a**) Western blot analysis for the detection of PSMB5 of a representative MM patient at baseline and at relapse. (**b**) Western blot analysis for the detection of LC3II of a representative MM patient at baseline and relapse. (**c**) Protein levels of PSMB5 at both time points of the whole patient cohort (*n* = 7) and of the controls. (**d**) Protein levels of LC3II at both time points of the whole patient cohort and of the controls (**c**,**d**) Each bar corresponds to the mean signal intensity per mg of total protein of each patient group, and the error bars correspond to the standard deviation. * corresponds to a *p*-value < 0.05.

A second cohort of patients (*n* = 7) (named the Relapse Cohort) was formed by individuals with paired bone marrow samples, both from initial diagnosis and from relapse. PSMB5 levels at baseline were calculated at 1.86 ± 0.34, whereas at relapse, they were found to be as low as 0.49 ± 0.23 (Figure 7a). The paired *t*-test indicated a statistically significant difference with a *p*-value = 0.036 (Figure 7c). PPA was also significantly diminished (*p*-value < 0.0001) at relapse, as shown in Figure 2c. Conversely, regarding autophagy, mean LC3II levels at baseline were found to be as low as 1.79 ± 0.56 and were increased at relapse up to 6.67 ± 1.23 (Figure 7b). A paired *t*-test verified the statistical significance with a *p*-value = 0.042 (Figure 7d). Western blots of both PSMB5 and LC3II from representative patients belonging to the Relapse Cohort are presented in Figure 7a,b.

Overall, the obtained values exhibited a high degree of proportionality in each individual patient at the two time points. These results are in line with those discussed previously regarding independent patient samples and further strengthen the notion that proteasome activity is decreased following exposure to PIs, while autophagy is activated. Thus, our findings indicate that myelomatous cell exposure to proteasome inhibitors leads to a significant decrease in the expression of the catalytic proteasomal subunit PSMB5, whereas LC3 expression is elevated. This observation probably indicates that when the UPS functionality is impaired, autophagy’s promotion acts as a counterbalance mechanism in order to sustain cell functionality and, hence, cell survival.

### 3.6. ROS Exhibit Negative Correlation with PSMB5 and Positive Correlation with LC3II

Additionally, the potential existence of an association between the proteasomal and autophagic activity of myelomatous plasma cells and the intracellular ROS levels was investigated.

**Figure A2:**
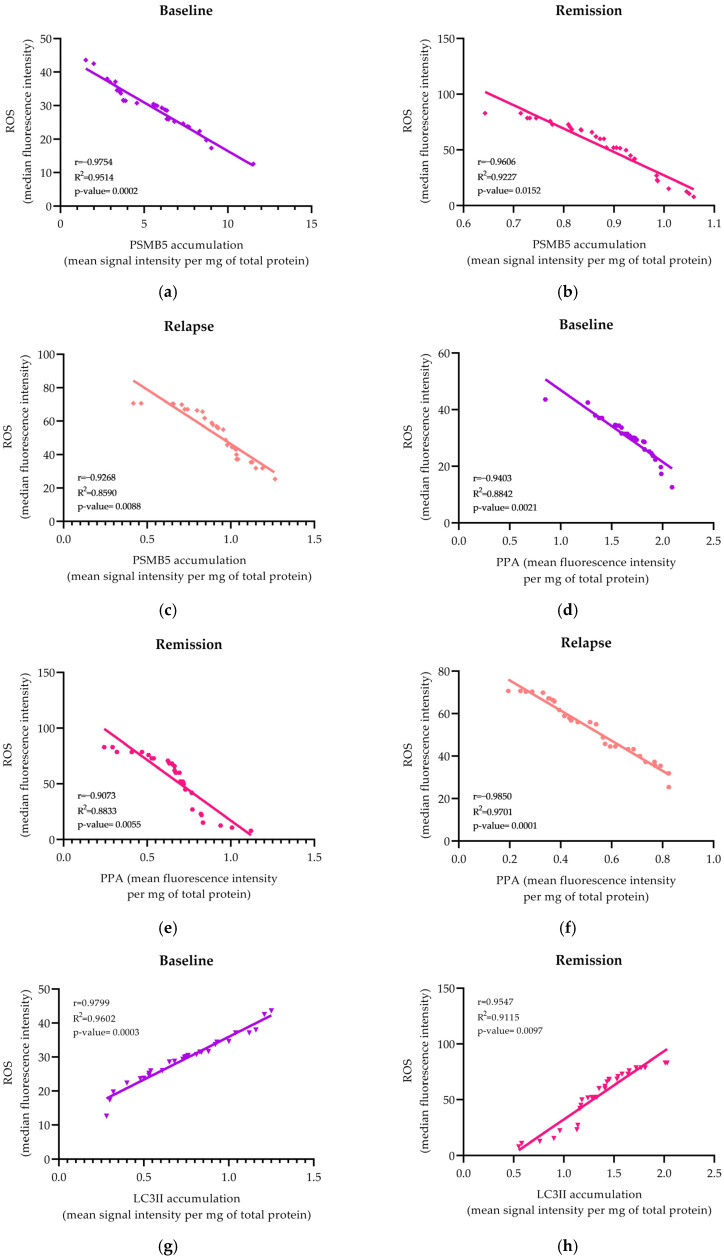

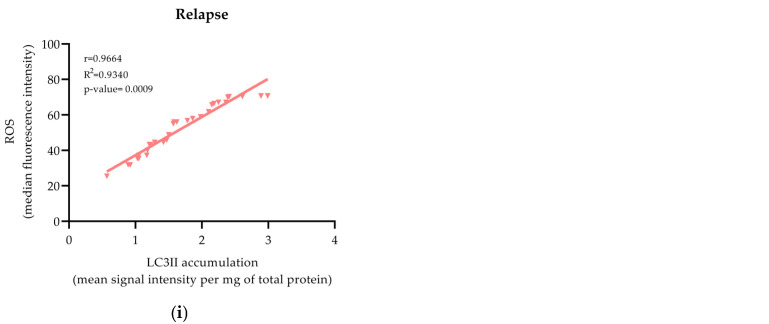
Pearson’s correlations. (**a**) PSMB5 and ROS coefficient at baseline, (**b**) at remission, and (**c**) at relapse. (**d**) PPA and ROS at baseline, (**e**) at remission, and (**f**) at relapse. (**g**) Correlation of LC3 and ROS level at baseline, (**h**) at remission, and (**i**) at relapse. (**a**–**i**) Pearson’s coefficient is annotated as (r), the correlation coefficient is annotated as (R^2^), and the *p*-values correspond to the linear regression analysis.

Using Pearson’s correlation, BMMC PSMB5 protein levels exhibited a strong (r < −0.5) negative correlation with ROS levels at baseline (r = −0.975, *p*-value = 0.002) (Figure A2a). The same finding was observed during remission (r = −0.961, *p*-value = 0.015) (Figure A2b) and at relapse (r = −0.927, *p*-value = 0.009) (Figure A2c). The same pattern was also observed regarding PPA and oxidative stress levels at all disease stages (baseline, first remission, and relapse). Correlation at baseline had an r = −0.940 (*p*-value = 0.002) (Figure A2d); at remission had an r = −0.907 (*p*-value = 0.006) (Figure A2e); and at relapse, the Pearson’s coefficient was r = −0.985 (*p*-value = 0.0001) (Figure A2f). For the aforementioned correlation analysis, the control sample was not included since our purpose was to study the progression of MM, and extrapolation would be impossible if both neoplastic and non-neoplastic cells were being studied together. Indeed, the PPA of the controls was found to be significantly lower than that of untreated MM patients (both as a sum of the sample findings and in each individual disease stage); however, oxidative stress levels of that group were the lowest among all.

On the other hand, LC3II accumulation exhibited a significant positive (r > 0.5) correlation with intracellular ROS levels in all three groups. LC3II and ROS at baseline had a Pearson’s coefficient of r = 0.980 (*p*-value = 0.0003) (Figure A2g); at remission, r = 0.955 (*p*-value = 0.010) (Figure A2h); and at relapse, r = 0.966 (*p*-value = 0.001) (Figure A2i). LC3II is a major autophagosome marker, and it is believed that elevated ROS levels may act as a trigger for the induction of autophagy in order to eliminate the presence of damaged molecules that induce further oxidative damage [70].

### 3.7. Baseline PSMB5 Protein Levels as a Prognostic Factor for Multiple Myeloma Patients

**Figure 8:**
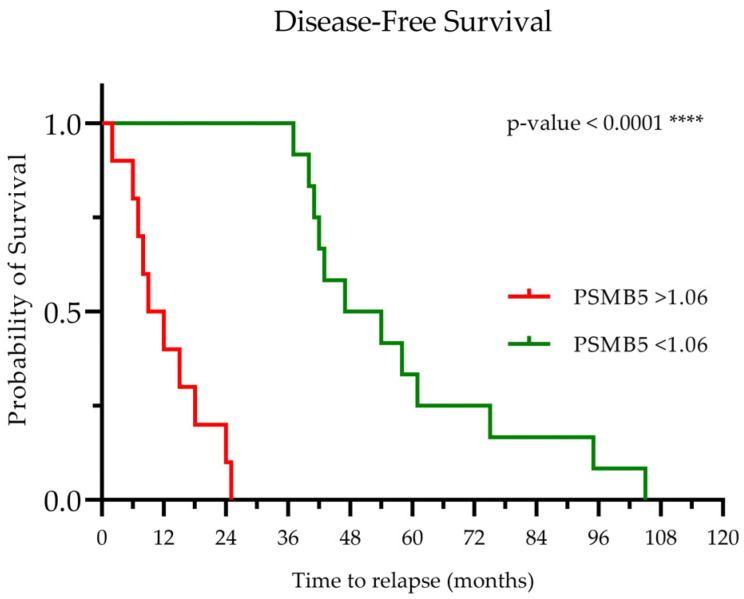
Kaplan–Meier estimates for disease-free survival in multiple myeloma patients. Patients were subcategorized into two groups according to their baseline PSMB5 protein accumulation level: red ribbon: patients with PSMB5 levels higher than 1.06; green ribbon: patients with PSMB5 levels at baseline lower than 1.06. A statistically significant difference was detected, and the group with a higher PSMB5 accumulation had a shorter disease-free survival than patients with PSMB5 accumulation lower than 1.06 (*p*-value < 0.0001). **** corresponds to a *p*-value < 0.0001.

Finally, survival analysis was performed to identify the potential importance of PSMB5 levels as a prognostic factor. MM patients studied at initial diagnosis were further subcategorized into two groups according to their PSMB5 subunit protein levels, corresponding to a higher or a lower accumulation. To form the groups, median PSMB5 subunit accumulation was used (for the patients whose data were available during the course of the study), which was calculated at 1.06 (mean signal intensity per mg of total protein). Thus, 1.06 was used as a cut-off value for the categorization mentioned above in order to study the time lapsed until disease relapse. Our results showed that patients who had PSMB5 levels higher than 1.06 at initial diagnosis relapsed earlier than patients who had PSMB5 levels lower than 1.06 (*p* < 0.001). The Kaplan–Meier overall survival analysis was calculated and is shown below (Figure 8).

We hypothesize that patients who exhibited a higher PSMB5 accumulation level at initial diagnosis might have a higher possibility of escaping the UPS inhibition under treatment with PIs. The cells resisting therapy could activate autophagy as a rescue mechanism, a cell function already acting as a competitive mechanism, with proteasome-dependent proteostasis and high ROS formation as a regulatory mechanism. Thus, myelomatous cells with activated autophagy could manage to escape the burdens set by PIs in the cellular processes (oxidative stress damages, cell cycle arrest, accumulation of damaged molecules, deregulation of cell metabolism, and the subsequent cell death), resulting in a shorter remission period, thus leading to faster relapse.

## 4. Discussion

In this study, we monitored the interplay between UPS and autophagy, as well as the role of oxidative stress in this balance, in whole BMMCs isolated from MM patients. Given the importance of the phenomenon, several studies have been previously published focusing on PI resistance, yet the underlying mechanisms have not yet been fully elucidated [12,21,42]. In the last two decades, researchers have thoroughly studied the signaling pathways of Bortezomib resistance and its genetic/epigenetic bases; however, only recently have they begun to publish holistic approaches regarding the interactions between various contributing pathogenetic mechanisms. The purpose of this study was to focus on the main proteostasis subsystems of BMMCs, namely UPS and autophagy, and to examine how they interact once resistance to PIs has been developed and whether changes of intracellular oxidative stress have a role and could be used as a marker. Our results imply that new therapeutic approaches for the disease could be designed, both at the preclinical and the clinical level.

PSMB5 was the main molecule that we investigated in this study, attempting to feature its importance in disease progression and to correlate its expression with changes in oxidative stress levels and autophagic flux. We observed that treatment with PI-containing regimens had a decreasing effect on PSMB5 subunit protein levels while concurrently, it increased the accumulation of LC3II protein. Since PSMB5 acts as a Bortezomib and other PIs’ main substrate, it is considered a key molecule for both susceptibility to PIs and the development of resistance [13,47]. Mutations in the *PSMB5* gene that reduce the affinity for association with Bortezomib have been recognized in many MM patients and in in vitro settings over the years [44,47,48]; however, not all resistant phenotypes carry *PSMB5* mutated alleles. Excessive PSMB5 synthesis can also enhance the plasma cell resistance to PIs by producing additional proteasomal subunits to compensate for the drug-bound inactivated ones [71]. Moreover, in cases in which the MDR phenotype has emerged, and MM plasma cells effectively efflux the inhibitor, *PSMB5* is not necessarily mutated. Nevertheless, mutated PSMB5 remains a cornerstone in PI resistance since it may be a result of selection due to Bortezomib treatment, and it largely affects MM response to next-generation PIs as well [44]. PSMB5 has been again examined in the past as a prognostic factor, and it has been demonstrated that PSMB5 deletions can resensitize PI-resistant cell lines to Bortezomib [72]. Recently, Robak et al. showed that whole-blood *PSMB5* mRNA, as well as *CXCR* mRNA, may have a prognostic value and could be used as independent predictors of PFS in myeloma patients [73]. These findings are in accordance with our results, which show that credit (whole) BMMC PSMB5 levels have a potential prognostic value as well. Besides PSMB5, other proteasome subunits, such as PSMB1, have also been proposed as prognostic markers by other researchers since they control the proteasomal sensitivity to PIs and, thus, the system’s flexibility and ability to develop escape mechanisms [72,74,75]. PSMB5 represents the main molecular target of Bortezomib and of other PIs, as it is the main 20S proteasome subunit bearing ChT-L activity; however, it is the sole subunit responsible for total ChT-L and PPA in general. In our study, PPA was studied independently from PSMB5 accumulation, and in all MM patients, a strong correlation between the two parameters was revealed. Of interest, in our control group, being (whole) BMMCs from non-infiltrated bone marrow, we observed similar PSMB5 expression levels. However, the PPA of the control samples was significantly lower. This finding might imply that in MM, additional ChT-L proteasomal subunits are active (such as immunoproteasome subunits), or post-transcriptional changes could have led to such a differential PPA activity. β5i (20S proteasome subunit beta-5i or proteasome subunit beta type-8, PSMB5 {or LMP7}) is another proteasome subunit with ChT-L activity, and it is a member of the immunoproteasome that replaces PSMB5 under treatment with interferon-γ [76]. PSMB8 activity is also affected by Bortezomib [76]; however, Shi et al., in 2020, created proteasome subunit-knockout myeloma cell lines and concluded that knocking out PSMB8 did not resensitize the cells to Bortezomib [72]. On the contrary, in the same study, knocking out PSMB5 was able to resensitize the cells, implying that PSMB8 may not be a major factor in Bortezomib-specific resistance [72]. Nevertheless, data regarding the PSMB8 role in MM are continuously increasing, and a novel PSMB8-specific inhibitor (M3258) has recently been discovered to exhibit increased in vitro efficacy in MM models [77,78,79]. On the other hand, it is also noteworthy that treatment-naïve MM patients of our study exhibited control-like PSMB5 levels. Many studies have reported that elevated PSMB5 levels have been observed in MM cell lines and patients [45,47]. However, this is not a necessity; Robak et al., in 2021, showed that PSMB5 expression levels of newly diagnosed MM patients and of healthy donors did not differ significantly [74]. As the disease progresses, resistance to PIs can be developed by PSMB5 upregulation and/or mutations that practically overcome the inhibition imposed by Bortezomib [44,46,47]. In cases of advanced resistance against PIs, myelomatous cells can abolish the expression of PSMB5 and upregulate subunits with a lower affinity for PIs or completely switch to proteasome-independent proteostasis pathways [48,52].

The role of autophagy in Bortezomib resistance is a relatively novel discovery, with several published studies proposing that it can act as a counterbalancing mechanism when UPS is rendered inadequate to cover the cell needs and catabolize the accumulating excessive, misfolded, or damaged proteins [43,52,53,55,80,81,82]. The molecular chaperone sequestosome-1 (SQSTM1/p62) is often cited as the link between UPS-dependent proteolysis and autophagy since it recognizes polyubiquitinated polypeptides and drives them to the autophagosomes for degradation. p62 has been reported to be overexpressed in Bortezomib-resistant cells, and the autophagic flux has also been found to be increased. According to Vu et al., another molecule with significant prognostic value in MM progression is TRIM44 (Tripartite Motif-Containing 44) [83]. They demonstrated how TRIM44 facilitates the oligomerization of p62 under high oxidative stress conditions, establishing a connection between the UPS, autophagy, and oxidative stress [83]. Additionally, overexpression of the autophagy–apoptosis regulator Beclin-1 (BECN1) has also been observed in cases of PI resistance, which is believed to promote survival by inducing apoptosis in an effort to recycle intracellular contents and thus mitigate pro-apoptosis signaling, which is caused by accumulated damaged or oxidized molecules [81]. Beclin-1 is known to interact with NEK2 (serine/threonine protein kinase, Nek2), a very important mediator of drug resistance, which has also been reported to be overexpressed. Xia et al., in 2020, created Beclin-1 knockdown clones of various MM cell lines, which were documented to be Bortezomib-sensitive [81]. They also demonstrated that inhibitors of autophagy, such as chloroquine, when co-administered with Bortezomib, can overcome autophagy-mediated Bortezomib resistance [81]. Both findings regarding autophagy markers, as well as more evidence regarding the role of autophagy in Bortezomib resistance, have also been confirmed by our group in prostate cancer cell lines used as models. Bortezomib-resistant clones of the PI-susceptible DU-145 and PC-3 cell lines were created, and the resistant phenotypes had clear evidence of autophagy upregulation and detachment from the UPS as the main proteostatic mechanism [43,57]. From a clinical perspective, a phase II clinical trial has also explored the potential use of chloroquine combined with Bortezomib and Cyclophosphamide to treat relapsed and refractory multiple myeloma and concluded that it could be an effective treatment approach [84]; in line with the above, other (phase I/II) trials studying hydroxychloroquine efficacy have made similar observations [85]. The transcription factor CRIP1 (cysteine-rich intestinal protein 1) was recently associated with poor outcomes in MM, and all findings suggest that it has a crucial role in the regulation of UPS and autophagy, emphasizing how the two mechanisms act supplementarily in the cell. CRIP1 was found to promote PI resistance by enhancing the maturation of the autophagosomes that act as substitutes for the impaired UPS [82]. In another study, it has been shown that autophagy inhibition by macrolide antibiotics (Clarithromycin) in Bortezomib-resistant multiple myeloma cell lines was able to suppress PI resistance and resensitize the cells to the induction of apoptosis [86]. All these findings suggest that autophagy should be considered as the next molecular target in the fight against PI resistance.

Oxidative stress acts as a major regulatory mechanism of autophagy, capable of inducing it when intracellular ROS levels are high but also suppressing it when oxidation is not a major problem by using negative inhibitory mechanisms [21,52,70]. In our study, we observed high intracellular ROS levels, both at remission and at relapse, which were inversely correlated with proteasome activity. Herein, we must mention that ROS detection was performed in both cell compartments, whole BMMCs, as well as in the myelomatous cell fraction (CD138+/CD38+), whose distinction was feasible, using flow cytometry. Thus, flow cytometry allowed us to investigate whether the double-positive, “strictly” malignant cells would exhibit findings similar to those of whole BMMCs. Indeed, both cell compartments, total BMMCs, and the double-positive subpopulation of myelomatous plasma cells exhibited results of high proportionality. Plasma cells from different patient groups followed the same ROS generation pattern, as did total BMMCs; however, it was to a higher extent. These findings are in accordance with the (well-established) oxidative-stress-inducing nature of Bortezomib [13,60,87]; however, our novelty was the simultaneous assessment of autophagosome creation by monitoring LC3II. We documented a direct, strongly negative correlation between PSMB5 accumulation and BMMC ROS levels of patients at remission and at relapse, while, at the same time, LC3II accumulation and ROS levels were positively correlated. These observations indicate that ROS generation is very intense and autophagic flux peaks since ROS formation acts as a signal that activates alternative pro-survival mechanisms. Given the significance of ROS elimination for cell survival and the high level of free radicals found inside the plasma cells, the expected finding would be a significantly higher expression of antioxidant defense enzymes, such as superoxide dismutases. Surprisingly, we did not document such an expression when we assessed the accumulation of MnSOD (or SOD2), but instead, SOD2 levels of all patients were lower than those of the controls and were decreasing even more as the disease progressed [69,87]. This finding possibly indicates that PI-resistant plasma cells could have evolved to downregulate SOD2 synthesis, thus being more susceptible to the accumulation of free radicals but also obtaining a very sensitive trigger mechanism for autophagy–apoptosis regulation. Therefore, from a clinical point of view, exploiting this novel, sensitive redox equilibrium could have beneficial effects in the elimination of resistant cells. It is also noteworthy that in the control group, even though relatively high levels of PSMB5 were detected, PPA remained significantly lower compared to other patient groups. This deviation was interpreted as potentially resulting from the still-functioning homeostatic mechanisms in the BMMCs of the controls. Nonetheless, the induction of ROS in treated MM patients might be multifactorial, being both the result of treatment with PIs and of other chemotherapeutic or targeted agents, as well as a direct consequence of genetic mutations that further deregulate the defense mechanisms.

It is important to highlight that in our study, whole BMMCs were used as a cell source for the evaluation of PSMB5, PPA, and LC3II levels, while only for ROS detection a plasma-cell-specific assay was feasible (CD138+/CD38+ gating in flow cytometry). This selection was the consequence of two main limitations: the large amount of bone marrow aspirate necessary to perform all the assays and their validation and the low viability rates of myelomatous plasma cells outside of the bone marrow microenvironment at the early stages of the disease. Outside the bone marrow, the stress levels increase, autophagic lysis of cell components is initiated, and cell death pathways are activated. Thus, such handling would significantly affect our results by inflating both ROS and autophagy marker levels. The use of whole BMMCs instead of purified CD138+/CD38+ plasma cells leads to a fluctuating plasma cell count from sample to sample. We showed that the percentage of plasma cells was relatively stable in each patient group; however, these percentages varied significantly between the different disease stages. Patients at their first remission had the lowest plasma cell count (~1%), meaning that about 99% of the analyzed BMMCs were not myelomatous cells. Although a strong association was observed between whole BMMCs and plasma cells when ROS were assessed, our results might be misleading if interpreted as a screening of myelomatous cells since our sample is a mix of myelomatous and non-myelomatous cells residing in the bone marrow. Nonetheless, the sample burden does not impose an important limitation regarding the results’ significance. Other studies have also investigated more “abstract”, less invasive, and, in general, more accessible proteasome-related molecular biomarkers to evaluate disease status and progression [74,88,89]. In 2004, Jakob et al. mentioned the use of circulating proteasome (c-proteasome) as a prognostic factor for survival in MM [88]. More recently, Robak et al., in 2021, investigated the expression of the *PSMB5* mRNA (among other genes) in whole-blood as a prognostic factor in MM [79]. The use of PSMB5 as a predictor of the development of PI resistance could have multiple potential benefits since it could allow for a more holistic approach to the disease. Moreover, the use of PSMB5 as a prognostic marker could also provide information about the activation of alternative cellular mechanisms and help clinicians further improve treatment regimens by incorporating novel agents to target these escape mechanisms. In our study, the identification of strong correlations between the parameters of proteasomal function, autophagy, and oxidative stress in the larger cellular pool of whole BMMCs implies that BMMCs can act as a representative cell population. Research for prognostic markers in MM ranges from liquid biopsies (that rely on the circulating plasma cells or MM-specific antigens to evaluate disease progression) to microenvironment studies (that attempt to evaluate disease status and aggressiveness by assessing interactions between myelomatous and non-myelomatous cells in the bone marrow) [90,91,92,93,94,95]. Our study lies somewhere in between those reference points, as it provides significant insight regarding both whole BMMCs condition in the bone marrow, as well as plasma-cell-specific characteristics like ROS generation. The function of the tumor microenvironment plays a huge role in disease progression, and the development of resistance in MM since bone marrow stromal cells (BMSCs) can significantly affect the growth of myelomatous BMMCs [96]. Autophagy is a major factor in this equilibrium since its activation in both BMSCs and malignant cells can release the pro-survival cytokine IL-6 (Interlekin-6) and other growth factors, thus allowing the disease to propagate [97]. Various potential biomarkers have been identified in this context, ranging from growth factors to extracellular matrix markers (ECM) and immunological response molecules like cytokines [98,99,100,101,102].

From the clinical point of view, our findings could significantly improve the management of MM patients following treatment with PIs (and especially Bortezomib), offering clinicians important insight into disease progression using PSMB5 as a marker. Integrating PSMB5, autophagy markers, and ROS testing in patient assessment could provide novel tools to estimate the emergence of resistance in PI-treated patients and predict outcomes. Testing for protein levels in the whole BMMCs instead of purified plasma cells (CD138+/CD38+) could be more feasible and practical than isolating plasma cells prior to cell analysis as it would save important time, resources, and biological material for other analyses. UPS and autophagy assaying might act as indicators of Bortezomib effectiveness and assist the physicians in determining whether a patient heads to disease relapse and a change of regimen is necessary. Our findings support the idea that autophagy is an important UPS substitute that potentially drives resistance and could be targeted to reverse PI resistance. Furthermore, the notion of treating MM with oxidative-stress-inducing regimens is highlighted as a way to overload the cell defense mechanisms. It is noteworthy that antimalaria drugs like chloroquine and hydroxychloroquine (as well as other autophagy inhibitors) are promising agents regarding the autophagy-related escape mechanism that have not yet entered clinical practice [54,85,86]. Of course, many limitations arise from the drug’s toxicity, which leaves only a very narrow therapeutic window [103,104]. Targeting the redox balance of the resistant cells by overwhelming their already sensitized defense system could also provide less toxic, better-tolerated alternatives to current chemotherapies. Even though our findings provide important insight into all these phenomena, more research is warranted to better understand the molecular basis of such interactions and the prognostic value of these molecules. More specifically, attention should be given to MM Bortezomib-resistant cells in conditions in which the antioxidant defense is overloaded and the autophagy is targeted with inhibitors. The signaling pathways activated in these conditions could be the next targets in PI-desensitized cells. Overall, our results also support the notion that targeting the proteasome’s main interactions could promote the use of PIs in malignancies in which UPS-targeting therapy remains ineffective, thus expanding our armory against other forms of cancer.

## 5. Conclusions

In this work, whole BMMCs were used to study the interplay between UPS, autophagy, and oxidative stress in MM patients at different stages of the disease and to investigate the potential prognostic value of main molecules like PSMB5 and LC3II in MM. Our findings suggest a negative correlation between PSMB5 and LC3II accumulation following the emergence of resistance to PIs, while an analogous negative correlation was observed between PSMB5 accumulation and intracellular plasma cell ROS levels. Our research underlines the significance of baseline BMMC PSMB5 levels as a prognostic marker for DFS in MM patients that could help to predict outcomes and disease aggressiveness. Since PI treatment leads to ROS accumulation and utilization of autophagy as an escape mechanism, pharmacological targeting of these parameters could also be a determinant factor in the fight against drug-resistant MM clones. The use of whole BMMCs instead of purified CD138+/CD38+ myelomatous cells suggests the application of a more rapid and cost-effective tool in clinical practice that could be used to estimate disease progression and PI treatment effectiveness. PSMB5, LC3II, and ROS (as well as other possible markers) could provide a fast and reliable tool to assess the bone marrow microenvironment regarding key cellular functions and assist in the design of future regimens.

## Figures and Tables

**Table 1:** Main patient characteristics regarding demographic, clinical, and treatment-related information (first-line treatment). Patients (*n* = 110) are stratified by disease status at the time of evaluation into three groups: (a) Initial diagnosis/baseline (pre-treatment) (*n* = 31), (b) first remission (*n* = 36), and (c) first relapse (*n* = 43). Basic characteristics of the control sample (*n* = 17) are also present.

	MM Patients	Initial Diagnosis	First Remission	First Relapse	Control Group
Number of Subjects, *n* (% of sample)	110	31 (28.2)	36 (32.7)	43 (39.1)	(*n* = 17)
**Demographic Characteristics**					
Age, Mean (SD)	70.6 (8.3)	70.6 (6.6)	68.3 (6.2)	71.6 (9.5)	69.4 (5.1)
Sex, *n* (% of Group)					
Male	65 (59.5)	24 (77.4)	19 (52.8)	22 (51.2)	7 (50.0%)
Female	45 (40.5)	7 (22.6)	17 (47.2)	21 (48.8)	7 (50.0%)
**Clinical Characteristics**					
Immune Classification, *n* (% of Sample)					
IgG Type	61 (55.4)	14 (12.7)	24 (21.8)	23 (20.9)	
κ light chain	28 (25.4)	13 (11.8)	3 (2.7)	12 (10.9)	
λ light chain	33 (30.0)	1 (0.9)	21 (19.1)	11 (10.0)	
IgA Type	29 (26.4)	13 (11.8)	6 (5.4)	10 (9.1)	
κ light chain	14 (12.8)	9 (8.2)	3 (2.7)	5 (4.5)	
λ light chain	15 (13.6)	4 (3.6)	3 (2.7)	5 (4.5)	
Light chains only	20 (17.2)	4 (3.6)	6 (5.4)	10 (9.1)	
κ light chain	6 (5.5)	0	6 (5.4)	8 (7.3)	
λ light chain	14 (12.7)	4 (3.6)	0	2 (1.8)	
ISS Stage, *n* (% of sample)					
I	34 (30.9)	8 (7.3)	11 (10.0)	15 (13.6)	
II	28 (25.5)	6 (5.4)	10 (9.1)	12 (10.9)	
III	48 (43.6)	17 (15.5)	15 (13.6)	16 (14.5)	
Plasma Cells, Range of %	16–32	35–60	1–5	15–35	0–1
**Treatment Characteristics**					
Regimen, *n* (% of Sample)					
VCD	57 (51.9)	25 (22.8)	20 (18.2)	16 (14.5)	
VCD->VRD	2 (1.8)	0	0	2 (1.8)	
VMP	18 (16.4)	3 (2.7)	7 (6.4)	8 (7.3)	
DVMP	5 (4.5)	1 (0.9)	0	4 (3.6)	
PAD	20 (18.2)	2 (1.8)	7 (6.4)	11 (10.0)	
PAD->Vel/Dex	2 (1.8)	0	0	2 (1.8)	
PAD->CAD	2 (1.8)	0	2 (1.8)	0	

Note: MM = multiple myeloma; ISS = International Staging System; SD = standard deviation; VCD = Velcade^®^ (Bortezomib) Cyclophosphamide Dexamethasone; VRD = Velcade^®^, Revlimid^®^ (Lenalidomide), and Dexamethasone; VMP = Velcade^®^, Melphalan, and Prednisone; DVMP = Daratumumab, Velcade^®^, Melphalan, and Prednisone; PAD = PS-341 (Bortezomib), Adriamycin (Doxorubicin), and Dexamethasone; Vel/Dex = Velcade^®^ and Dexamethasone; CAD = Cyclophosphamide, Adriamycin, and Dexamethasone.

## Data Availability

The data presented in this study are available on request from the corresponding author.

## Appendix A

Additional patient clinical characteristics.

**Table A1:** A set of additional patient demographic and clinical characteristics. The patient group is composed of 110 subjects, and the control group is composed of 17 subjects.

	MM Patients	Control Group
	*n* = 110	*n* = 17
**Demographic Characteristics**		
Age, Mean (SD)	70.6 (8.32)	69.4 (5.11)
Sex, *n* (% of Group)		
Male	65 (59.5)	7 (50.0%)
Female	45 (40.5)	7 (50.0%)
**Clinical Characteristics**		
Poor Prognosis Cytogenetics ^1^, *n* (% of Sample ^2^)		
Yes	22 (22.7)	
No	73 (77.3)	
Renal Impairment ^3^, *n* (% of Sample)		
Yes	10 (9.0)	
No	100 (91.0)	
Anemia ^4^		
Yes	42 (38.0)	
No	68 (62.0)	
Osteolytic Bone Disease, *n* (% of Sample)		
Yes	74 (67.2)	
No	36 (32.8)	

Note: ^1^. Poor prognosis cytogenetics: IGH translocations t(4;14) and t(14;16) and t(14;20), del(17q), p53 mutation, 1q gain/amplification, and 1p deletion. ^2^. In the case of cytogenetics testing, 97 out of 110 patients were screened. The percentage in that cell refers to the subset of tested patients rather than the whole sample. ^3^. Glomerular filtration rate (GFR) <30 mL/min/1.73 mm^2^; ^4^ Anemia: hemoglobin < 11 g/dL.

## Appendix B

Linear correlation plots.
